# Current status of community resources and priorities for weed genomics research

**DOI:** 10.1186/s13059-024-03274-y

**Published:** 2024-05-27

**Authors:** Jacob Montgomery, Sarah Morran, Dana R. MacGregor, J. Scott McElroy, Paul Neve, Célia Neto, Martin M. Vila-Aiub, Maria Victoria Sandoval, Analia I. Menéndez, Julia M. Kreiner, Longjiang Fan, Ana L. Caicedo, Peter J. Maughan, Bianca Assis Barbosa Martins, Jagoda Mika, Alberto Collavo, Aldo Merotto, Nithya K. Subramanian, Muthukumar V. Bagavathiannan, Luan Cutti, Md. Mazharul Islam, Bikram S. Gill, Robert Cicchillo, Roger Gast, Neeta Soni, Terry R. Wright, Gina Zastrow-Hayes, Gregory May, Jenna M. Malone, Deepmala Sehgal, Shiv Shankhar Kaundun, Richard P. Dale, Barend Juan Vorster, Bodo Peters, Jens Lerchl, Patrick J. Tranel, Roland Beffa, Alexandre Fournier-Level, Mithila Jugulam, Kevin Fengler, Victor Llaca, Eric L. Patterson, Todd A. Gaines

**Affiliations:** 1 Department of Agricultural Biology, Colorado State University, 1177 Campus Delivery, Fort Collins, CO 80523 USA; 2 Protecting Crops and the Environment, Rothamsted Research, Harpenden, Hertfordshire UK; 3 Department of Crop, Soil, and Environmental Sciences, Auburn University, Auburn, AL USA; 4 Department of Plant and Environmental Sciences, University of Copenhagen, Taastrup, Denmark; 5 IFEVA-Conicet-Department of Ecology, University of Buenos Aires, Buenos Aires, Argentina; 6 Department of Ecology, Faculty of Agronomy, University of Buenos Aires, Buenos Aires, Argentina; 7 Department of Botany, The University of British Columbia, Vancouver, BC Canada; 8 Institute of Crop Sciences, Zhejiang University, Hangzhou, China; 9 Department of Biology, University of Massachusetts Amherst, Amherst, MA USA; 10 Department of Plant and Wildlife Sciences, Brigham Young University, Provo, UT USA; 11 Bayer AG, Weed Control Research, Frankfurt, Germany; 12 Department of Crop Sciences, Federal University of Rio Grande Do Sul, Porto Alegre, Rio Grande Do Sul Brazil; 13 Department of Soil and Crop Sciences, Texas A&M University, College Station, TX USA; 14 Department of Plant, Soil and Microbial Sciences, Michigan State University, East Lansing, MI USA; 15 Department of Agronomy, Kansas State University, Manhattan, KS USA; 16 Department of Plant Pathology, Kansas State University, Manhattan, KS USA; 17 Crop Protection Discovery and Development, Corteva Agriscience, Indianapolis, IN USA; 18 Genome Center of Excellence, Corteva Agriscience, Johnston, IA USA; 19 School of Agriculture, Food and Wine, University of Adelaide, Glen Osmond, South Australia Australia; 20 Jealott’s Hill International Research Centre, Syngenta Ltd, Bracknell, Berkshire UK; 21 Department of Plant and Soil Sciences, University of Pretoria, Pretoria, South Africa; 22 BASF SE, Ludwigshafen Am Rhein, Germany; 23 Department of Crop Sciences, University of Illinois, Urbana, IL USA; 24 Senior Scientist Consultant, Herbicide Resistance Action Committee / CropLife International, Liederbach, Germany; 25 School of BioSciences, University of Melbourne, Parkville, VIC Australia

**Keywords:** Weed science, Reference genomes, Rapid adaptation, Herbicide resistance, Public resources

## Abstract

Weeds are attractive models for basic and applied research due to their impacts on agricultural systems and capacity to swiftly adapt in response to anthropogenic selection pressures. Currently, a lack of genomic information precludes research to elucidate the genetic basis of rapid adaptation for important traits like herbicide resistance and stress tolerance and the effect of evolutionary mechanisms on wild populations. The International Weed Genomics Consortium is a collaborative group of scientists focused on developing genomic resources to impact research into sustainable, effective weed control methods and to provide insights about stress tolerance and adaptation to assist crop breeding.

**Supplementary Information:**

The online version contains supplementary material available at 10.1186/s13059-024-03274-y.

## Background

Each year globally, agricultural producers and landscape managers spend billions of US dollars [1, 2] and countless hours attempting to control weedy plants and reduce their adverse effects. These management methods range from low-tech (e.g., pulling plants from the soil by hand) to extremely high-tech (e.g., computer vision-controlled spraying of herbicides). Regardless of technology level, effective control methods serve as strong selection pressures on weedy plants and often result in rapid evolution of weed populations resistant to such methods [3–7]. Thus, humans and weeds have been locked in an arms race, where humans develop new or improved control methods and weeds adapt and evolve to circumvent such methods.

Applying genomics to weed science offers a unique opportunity to study rapid adaptation, epigenetic responses, and examples of evolutionary rescue of diverse weedy species in the face of widespread and powerful selective pressures. Furthermore, lessons learned from these studies may also help to develop more sustainable control methods and to improve crop breeding efforts in the face of our ever-changing climate. While other research fields have used genetics and genomics to uncover the basis of many biological traits [8–11] and to understand how ecological factors affect evolution [12, 13], the field of weed science has lagged behind in the development of genomic tools essential for such studies [14]. As research in human and crop genetics pushes into the era of pangenomics (i.e., multiple chromosome scale genome assemblies for a single species [15, 16]), publicly available genomic information is still lacking or severely limited for the majority of weed species. Recent reviews of current weed genomes identified 26 [17] and 32 weed species with sequenced genomes [18]—many assembled to a sub-chromosome level.

Here, we summarize the current state of weed genomics, highlighting cases where genomics approaches have successfully provided insights on topics such as population genetic dynamics, genome evolution, and the genetic basis of herbicide resistance, rapid adaptation, and crop dedomestication. These highlighted investigations all relied upon genomic resources that are relatively rare for weedy species. Throughout, we identify additional resources that would advance the field of weed science and enable further progress in weed genomics. We then introduce the International Weed Genomics Consortium (IWGC), an open collaboration among researchers, and describe current efforts to generate these additional resources.

## Evolution of weediness: potential research utilizing weed genomics tools

Weeds can evolve from non-weed progenitors through wild colonization, crop de-domestication, or crop-wild hybridization [19]. Because the time span in which weeds have evolved is necessarily limited by the origins of agriculture, these non-weed relatives often still exist and can be leveraged through population genomic and comparative genomic approaches to identify the adaptive changes that have driven the evolution of weediness. The ability to rapidly adapt, persist, and spread in agroecosystems are defining features of weedy plants, leading many to advocate agricultural weeds as ideal candidates for studying rapid plant adaptation [20–23]. The insights gained from applying plant ecological approaches to the study of rapid weed adaptation will move us towards the ultimate goals of mitigating such adaptation and increasing the efficacy of crop breeding and biotechnology [14].

### Biology and ecological genomics of weeds

The impressive community effort to create and maintain resources for *Arabidopsis thaliana* ecological genomics provides a motivating example for the emerging study of weed genomics [24–27]. *Arabidopsis thaliana* was the first flowering plant species to have its genome fully sequenced [28] and rapidly became a model organism for plant molecular biology. As weedy genomes become available, collection, maintenance, and resequencing of globally distributed accessions of these species will help to replicate the success found in ecological studies of *A. thaliana* [29–35]. Evaluation of these accessions for traits of interest to produce large phenomics data sets (as in [36–40]) enables genome-wide association studies and population genomics analyses aimed at dissecting the genetic basis of variation in such traits [41]. Increasingly, these resources (e.g. the 1001 genomes project [29]) have enabled *A. thaliana* to be utilized as a model species to explore the eco-evolutionary basis of plant adaptation in a more realistic ecological context. Weedy species should supplement lessons in eco-evolutionary genomics learned from these experiments in *A. thaliana*.

Untargeted genomic approaches for understanding the evolutionary trajectories of populations and the genetic basis of traits as described above rely on the collection of genotypic information from across the genome of many individuals. While whole-genome resequencing accomplishes this requirement and requires no custom methodology, this approach provides more information than is necessary and is prohibitively expensive in species with large genomes. Development and optimization of genotype-by-sequencing methods for capturing reduced representations of newly sequence genomes like those described by [42–44] will reduce the cost and computational requirements of genetic mapping and population genetic experiments. Most major weed species do not currently have protocols for stable transformation, a key development in the popularity of *A. thaliana* as a model organism and a requirement for many functional genomic approaches. Functional validation of genes/variants believed to be responsible for traits of interest in weeds has thus far relied on transiently manipulating endogenous gene expression [45, 46] or ectopic expression of a transgene in a model system [47–49]. While these methods have been successful, few weed species have well-studied viral vectors to adapt for use in virus induced gene silencing. Spray induced gene silencing is another potential option for functional investigation of candidate genes in weeds, but more research is needed to establish reliable delivery and gene knockdown [50]. Furthermore, traits with complex genetic architecture divergent between the researched and model species may not be amenable to functional genomic approaches using transgenesis techniques in model systems. Developing protocols for reduced representation sequencing, stable transformation, and gene editing/silencing in weeds will allow for more thorough characterization of candidate genetic variants underlying traits of interest.

Beyond rapid adaptation, some weedy species offer an opportunity to better understand co-evolution, like that between plants and pollinators and how their interaction leads to the spread of weedy alleles (Additional File 1: Table S1). A suite of plant–insect traits has co-evolved to maximize the attraction of the insect pollinator community and the efficiency of pollen deposition between flowers ensuring fruit and seed production in many weeds [51, 52]. Genetic mapping experiments have identified genes and genetic variants responsible for many floral traits affecting pollinator interaction including petal color [53–56], flower symmetry and size [57–59], and production of volatile organic compounds [60–62] and nectar [63–65]. While these studies reveal candidate genes for selection under co-evolution, herbicide resistance alleles may also have pleiotropic effects on the ecology of weeds [66], altering plant-pollinator interactions [67]. Discovery of genes and genetic variants involved in weed-pollinator interaction and their molecular and environmental control may create opportunities for better management of weeds with insect-mediated pollination. For example, if management can disrupt pollinator attraction/interaction with these weeds, the efficiency of reproduction may be reduced.

A more complete understanding of weed ecological genomics will undoubtedly elucidate many unresolved questions regarding the genetic basis of various aspects of weediness. For instance, when comparing populations of a species from agricultural and non-agricultural environments, is there evidence for contemporary evolution of weedy traits selected by agricultural management or were “natural” populations pre-adapted to agroecosystems? Where there is differentiation between weedy and natural populations, which traits are under selection and what is the genetic basis of variation in those traits? When comparing between weedy populations, is there evidence for parallel versus non-parallel evolution of weediness at the phenotypic and genotypic levels? Such studies may uncover fundamental truths about weediness. For example, is there a common phenotypic and/or genotypic basis for aspects of weediness among diverse weed species? The availability of characterized accessions and reference genomes for species of interest are required for such studies but only a few weedy species have these resources developed.

### Population genomics

Weed species are certainly fierce competitors, able to outcompete crops and endemic species in their native environment, but they are also remarkable colonizers of perturbed habitats. Weeds achieve this through high fecundity, often producing tens of thousands of seeds per individual plant [68–70]. These large numbers in terms of demographic population size often combine with outcrossing reproduction to generate high levels of diversity with local effective population sizes in the hundreds of thousands [71, 72]. This has two important consequences: weed populations retain standing genetic variation and generate many new mutations, supporting weed success in the face of harsh control. The generation of genomic tools to monitor weed populations at the molecular level is a game-changer to understanding weed dynamics and precisely testing the effect of artificial selection (i.e., management) and other evolutionary mechanisms on the genetic make-up of populations.

Population genomic data, without any environmental or phenotypic information, can be used to scan the genomes of weed and non-weed relatives to identify selective sweeps, pointing at loci supporting weed adaptation on micro- or macro-evolutionary scales. Two recent within-species examples include weedy rice, where population differentiation between weedy and domesticated populations was used to identify the genetic basis of weedy de-domestication [73], and common waterhemp, where consistent allelic differences among natural and agricultural collections resolved a complex set of agriculturally adaptive alleles [74, 75]. A recent comparative population genomic study of weedy barnyardgrass and crop millet species has demonstrated how inter-specific investigations can resolve the signatures of crop and weed evolution [76] (also see [77] for a non-weed climate adaptation example). Multiple sequence alignments across numerous species provide complementary insight into adaptive convergence over deeper timescales, even with just one genomic sample per species (e.g., [78, 79]). Thus, newly sequenced weed genomes combined with genomes available for closely related crops (outlined by [14, 80]) and an effort to identify other non-weed wild relatives will be invaluable in characterizing the genetic architecture of weed adaptation and evolution across diverse species.

Weeds experience high levels of genetic selection, both artificial in response to agricultural practices and particularly herbicides, and natural in response to the environmental conditions they encounter [81, 82]. Using genomic analysis to identify loci that are the targets of selection, whether natural or artificial, would point at vulnerabilities that could be leveraged against weeds to develop new and more sustainable management strategies [83]. This is a key motivation to develop genotype-by-environment association (GEA) and selective sweep scan approaches, which allow researchers to resolve the molecular basis of multi-dimensional adaptation [84, 85]. GEA approaches, in particular, have been widely used on landscape-wide resequencing collections to determine the genetic basis of climate adaptation (e.g., [27, 86, 87]), but have yet to be fully exploited to diagnose the genetic basis of the various aspects of weediness [88]. Armed with data on environmental dimensions of agricultural settings, such as focal crop, soil quality, herbicide use, and climate, GEA approaches can help disentangle how discrete farming practices have influenced the evolution of weediness and resolve broader patterns of local adaptation across a weed’s range. Although non-weedy relatives are not technically required for GEA analyses, inclusion of environmental and genomic data from weed progenitors can further distinguish genetic variants underpinning weed origins from those involved in local adaptation.

New weeds emerge frequently [89], either through hybridization between species as documented for sea beet (*Beta vulgaris* ssp. *maritima)* hybridizing with crop beet to produce progeny that are well adapted to agricultural conditions [90–92], or through the invasion of alien species that find a new range to colonize. Biosecurity measures are often in place to stop the introduction of new weeds; however, the vast scale of global agricultural commodity trade precludes the possibility of total control. Population genomic analysis is now able to measure gene flow between populations [74, 93–95] and identify populations of origin for invasive species including weeds [96–98]. For example, the invasion route of the pest fruitfly *Drosophila suzukii* from Eastern Asia to North America and Europe through Hawaii was deciphered using Approximate Bayesian Computation on high-throughput sequencing data from a global sample of multiple populations [99]. Genomics can also be leveraged to predict invasion rather than explain it. The resequencing of a global sample of common ragweed (*Ambrosia artemisiifolia* L.) elucidated a complex invasion route whereby Europe was invaded by multiple introductions of American ragweed that hybridized in Europe prior to a subsequent introduction to Australia [100, 101]. In this context, the use of genomically informed species distribution models helps assess the risk associated with different source populations, which in the case of common ragweed, suggests that a source population from Florida would allow ragweed to invade most of northern Australia [102]. Globally coordinated research efforts to understand potential distribution models could support the transformation of biosecurity from perspective analysis towards predictive risk assessment.

### Herbicide resistance and weed management

Herbicide resistance is among the numerous weedy traits that can evolve in plant populations exposed to agricultural selection pressures. Over-reliance on herbicides to control weeds, along with low diversity and lack of redundancy in weed management strategies, has resulted in globally widespread herbicide resistance [103]. To date, 272 herbicide-resistant weed species have been reported worldwide, and at least one resistance case exists for 21 of the 31 existing herbicide sites of action [104]—significantly limiting chemical weed control options available to agriculturalists. This limitation of control options is exacerbated by the recent lack of discovery of herbicides with new sites of action [105].

Herbicide resistance may result from several different physiological mechanisms. Such mechanisms have been classified into two main groups, target-site resistance (TSR) [4, 106] and non-target-site resistance (NTSR) [4, 107]. The first group encompasses changes that reduce binding affinity between a herbicide and its target [108]. These changes may provide resistance to multiple herbicides that have a common biochemical target [109] and can be effectively managed through mixture and/or rotation of herbicides targeting different sites of action [110]. The second group (NTSR), includes alterations in herbicide absorption, translocation, sequestration, and/or metabolism that may lead to unpredictable pleotropic cross-resistance profiles where structurally and functionally diverse herbicides are rendered ineffective by one or more genetic variant(s) [47]. This mechanism of resistance threatens not only the efficacy of existing herbicidal chemistries, but also ones yet to be discovered. While TSR is well understood because of the ease of identification and molecular characterization of target site variants, NTSR mechanisms are significantly more challenging to research because they are often polygenic, and the resistance causing element(s) are not well understood [111].

Improving the current understanding of metabolic NTSR mechanisms is not an easy task, since genes of diverse biochemical functions are involved, many of which exist as extensive gene families [109, 112]. Expression changes of NTSR genes have been implicated in several resistance cases where the protein products of the genes are functionally equivalent across sensitive and resistant plants, but their relative abundance leads to resistance. Thus, regulatory elements of NTSR genes have been scrutinized to understand their role in NTSR mechanisms [113]. Similarly, epigenetic modifications have been hypothesized to play a role in NTSR, with much remaining to be explored [114–116]. Untargeted approaches such as genome-wide association, selective sweep scans, linkage mapping, RNA-sequencing, and metabolomic profiling have proven helpful to complement more specific biochemical- and chemo-characterization studies towards the elucidation of NTSR mechanisms as well as their regulation and evolution [47, 117–124]. Even in cases where resistance has been attributed to TSR, genetic mapping approaches can detect other NTSR loci contributing to resistance (as shown by [123]) and provide further evidence for the role of TSR mutations across populations. Knowledge of the genetic basis of NTSR will aid the rational design of herbicides by screening new compounds for interaction with newly discovered NTSR proteins during early research phases and by identifying conserved chemical structures that interact with these proteins that should be avoided in small molecule design.

Genomic resources can also be used to predict the protein structure for novel herbicide target site and metabolism genes. This will allow for prediction of efficacy and selectivity for new candidate herbicides in silico to increase herbicide discovery throughput as well as aid in the design and development of next-generation technologies for sustainable weed management. Proteolysis targeting chimeras (PROTACs) have the potential to bind desired targets with great selectivity and degrade proteins by utilizing natural protein ubiquitination and degradation pathways within plants [125]. Spray-induced gene silencing in weeds using oligonucleotides has potential as a new, innovative, and sustainable method for weed management, but improved methods for design and delivery of oligonucleotides are needed to make this technique a viable management option [50]. Additionally, success in the field of pharmaceutical drug discovery in the development of molecules modulating protein–protein interactions offers another potential avenue towards the development of herbicides with novel targets [126, 127]. High-quality reference genomes allow for the design of new weed management technologies like the ones listed here that are specific to—and effective across—weed species but have a null effect on non-target organisms.

### Comparative genomics and genome biology

The genomes of weed species are as diverse as weed species themselves. Weeds are found across highly diverged plant families and often have no phylogenetically close model or crop species relatives for comparison. On all measurable metrics, weed genomes run the gamut. Some have smaller genomes like *Cyperus* spp. (~ 0.26 Gb) while others are larger, such as *Avena fatua* (~ 11.1 Gb) (Table 1). Some have high heterozygosity in terms of single-nucleotide polymorphisms, such as the *Amaranthus* spp., while others are primarily self-pollinated and quite homozygous, such as *Poa annua* [128, 129]. Some are diploid such as *Conyza canadensis* and *Echinochloa haploclada* while others are polyploid such as *C. sumetrensis*, *E. crus-galli*, and *E. colona* [76]. The availability of genomic resources in these diverse, unexplored branches of the tree of life allows us to identify consistencies and anomalies in the field of genome biology.

**Table 1:** Genome assemblies of 31 weed species completed or ongoing by the International Weed Genomics Consortium. All completed genomes are platinum assembly quality, defined as having chromosome-length scaffolds (i.e., 1–3 scaffolds per chromosome) for the assembly, unless indicated by *. Genome size estimated from flow cytometry or published references as indicated. + indicates that verification is currently in progress for cytogenetic information

Scientific name	Common name	Haplotypes in Assembly	Anticipated Availability Date	Ploidy	*x*	*n*	Genome size estimate (Gbp)
*Amaranthus hybridus*	Smooth pigweed	1; Previous version [130]	July 2024	Diploid	16	16	0.509 [131]
*Amaranthus palmeri*	Palmer amaranth	2; Previous version [130]	July 2024	Diploid	17	17	0.445 [132]
*Amaranthus retroflexus*	Redroot pigweed	1	July 2024	Diploid	17	17	0.592 [132]
*Amaranthus tuberculatus*	Common waterhemp	2; Previous version [130]	June 2024	Diploid	16	16	0.694 [132]
*Ambrosia artemisiifolia*	Common ragweed	2; Previous versions [100, 133]	December 2024	Diploid [134, 135]	18	18	1.152 [136]
*Ambrosia trifida*	Giant ragweed	2; Previous version [133]	July 2024	Diploid [134]	12	12	1.872 [137]
*Apera spica-venti*	Loose silkybent	2	October 2024	Diploid	7	7	4.622
*Avena fatua*	Wild oat	1	October 2024	Hexaploid (Additional file 2: Fig S1)	7	21	11.248
*Chenopodium album*	Common lambsquarters	1	July 2024	Hexaploid	9	27	1.59
*Cirsium arvense*	Canada thistle	2	December 2024	Diploid	17	17	1.415
*Convolvulus arvensis*	Field bindweed		In progress	Diploid^+^	12^+^	12^+^	0.652 [136]
*Conyza bonariensis (Erigeron bonariensis)*	Hairy fleabane	2	December 2024	Hexaploid [138]	9	27	2.043 [139]
*Conyza sumatrensis (Erigeron sumatrensis)*	Sumatran fleabane	1	October 2024	Hexaploid	9	27	1.874
*Cyperus esculentus*	Yellow nutsedge	2; Previous version [140]	December 2024	Diploid	54	54	0.588 [141]
*Cyperus rotundus*	Purple nutsedge	2	December 2024	Diploid	54	54	0.49 [141]
*Digitaria insularis*	Sourgrass	1	December 2024	Tetraploid	9	18	1.529
*Digitaria ischaemum*	Hairy crabgrass	1	December 2024	Tetraploid	9	18	0.625
*Echinochloa colona*	Junglerice (weedy genotype)	1; See crop genotype assembly by [76]	October 2024	Hexaploid	9	27	1.372 [141]
*Euphorbia esula*	Leafy spurge	2	December 2024	Hexaploid^+^ (based on [142, 143])	10^+^	60^+^	2.3 [144]
*Euphorbia heterophylla*	Wild poinsettia	2	December 2024	Diploid [145]	14	14	Unknown, in progress
*Leptochloa chinensis*	Chinese sprangletop	2; See also [146]	December 2024	Tetraploid	10	20	0.454
*Lolium rigidum*	Annual ryegrass	2; See also [147]	November 2024	Diploid (Additional file 2: Fig S2)	7	7	2.41
*Orobanche cernua*	Nodding broomrape		In progress	Diploid	19	19	1.421 [148]
*Orobanche crenata*	Crenate broomrape		In progress	Diploid	19	19	2.787 [148]
*Orobanche minor*	Small broomrape		In progress	Diploid	19	19	1.792 [148]
*Parthenium hysterophorus*	Ragweed parthenium	2	December 2024	Diploid [149]	17	17	Unknown, in progress
*Phalaris minor*	Little seed canary grass	1	November 2024	Tetraploid (Additional file 2: Fig S3)	7	14	5.851
*Raphanus raphanistrum	Wild radish	Previous versions [150, 151]	In progress	Diploid	9	9	0.515 [150]
*Salsola tragus*	Russian thistle	2	July 2024	Tetraploid (Additional file 2: Fig S4)	9	18	1.319
*Sorghum halepense	Johnsongrass	2	October 2024	Tetraploid	10	20	1.752
*Verbascum blattaria*	Moth mullein	1	December 2024	Diploid	15	15	0.344 [152]

The weed genomes published so far have focused mainly on weeds of agronomic crops, and studies have revolved around their ability to resist key herbicides. For example, genomic resources were vital in the elucidation of herbicide resistance cases involving target site gene copy number variants (CNVs). Gene CNVs of 5-enolpyruvylshikimate-3-phosphate synthase (*EPSPS*) have been found to confer resistance to the herbicide glyphosate in diverse weed species. To date, nine species have independently evolved *EPSPS* CNVs, and species achieve increased *EPSPS* copy number via different mechanisms [153]. For instance, the *EPSPS* CNV in *Bassia scoparia* is caused by tandem duplication, which is accredited to transposable element insertions flanking *EPSPS* and subsequent unequal crossing over events [154, 155]. In *Eleusine indica*, a *EPSPS* CNV was caused by translocation of the *EPSPS* locus into the subtelomere followed by telomeric sequence exchange [156]. One of the most fascinating genome biology discoveries in weed science has been that of extra-chromosomal circular DNAs (eccDNAs) that harbor the *EPSPS* gene in the weed species *Amaranthus palmeri* [157, 158]. In this case, the eccDNAs autonomously replicate separately from the nuclear genome and do not reintegrate into chromosomes, which has implications for inheritance, fitness, and genome structure [159]. These discoveries would not have been possible without reference assemblies of weed genomes, next-generation sequencing, and collaboration with experts in plant genomics and bioinformatics.

Another question that is often explored with weedy genomes is the nature and composition of gene families that are associated with NTSR. Gene families under consideration often include cytochrome P450s (CYPs), glutathione-*S*-transferases (GSTs), ABC transporters, etc. Some questions commonly considered with new weed genomes include how many genes are in each of these gene families, where are they located, and which weed accessions and species have an over-abundance of them that might explain their ability to evolve resistance so rapidly [76, 146, 160, 161]? Weed genome resources are necessary to answer questions about gene family expansion or contraction during the evolution of weediness, including the role of polyploidy in NTSR gene family expansion as explored by [162].

### Translational research and communication with weed management stakeholders

Whereas genomics of model plants is typically aimed at addressing fundamental questions in plant biology, and genomics of crop species has the obvious goal of crop improvement, goals of genomics of weedy plants also include the development of more effective and sustainable strategies for their management. Weed genomic resources assist with these objectives by providing novel molecular ecological and evolutionary insights from the context of intensive anthropogenic management (which is lacking in model plants), and offer knowledge and resources for trait discovery for crop improvement, especially given that many wild crop relatives are also important agronomic weeds (e.g., [163]). For instance, crop-wild relatives are valuable for improving crop breeding for marginal environments [164]. Thus, weed genomics presents unique opportunities and challenges relative to plant genomics more broadly. It should also be noted that although weed science at its core is an applied discipline, it draws broadly from many scientific disciplines such as, plant physiology, chemistry, ecology, and evolutionary biology, to name a few. The successful integration of weed-management strategies, therefore, requires extensive collaboration among individuals collectively possessing the necessary expertise [165].

With the growing complexity of herbicide resistance management, practitioners are beginning to recognize the importance of understanding resistance mechanisms to inform appropriate management tactics [14]. Although weed science practitioners do not need to understand the technical details of weed genomics, their appreciation of the power of weed genomics—together with their unique insights from field observations—will yield novel opportunities for applications of weed genomics to weed management. In particular, combining field management history with information on weed resistance mechanisms is expected to provide novel insights into evolutionary trajectories (e.g. [6, 166]), which can be utilized for disrupting evolutionary adaptation. It can be difficult to obtain field history information from practitioners, but developing an understanding among them of the importance of such information can be invaluable.

## Development of weed genomics resources by the IWGC

**Fig. 1:**
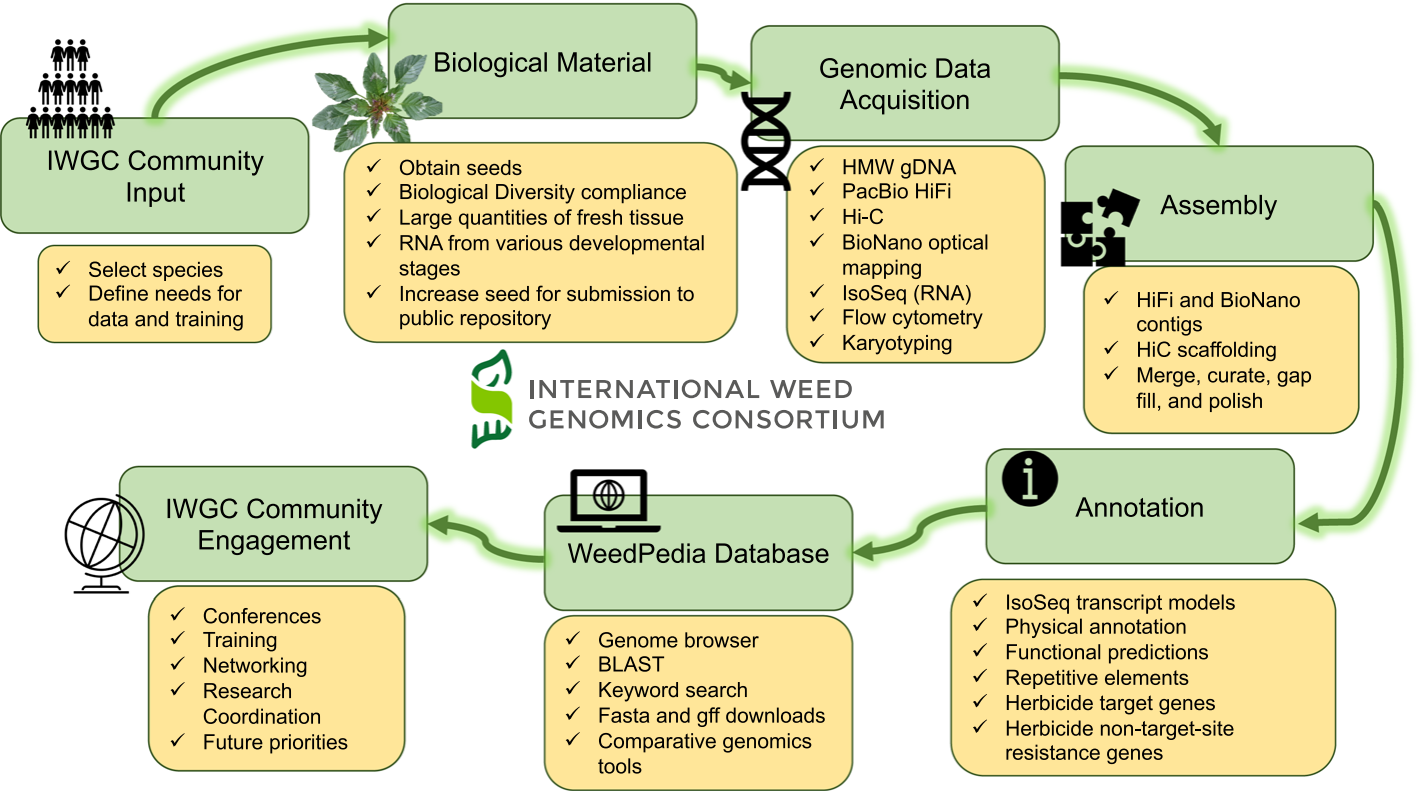
The International Weed Genomics Consortium (IWGC) collected input from the weed genomics community to develop plans for weed genome sequencing, annotation, user-friendly genome analysis tools, and community engagement

Weed genomics is a fast-growing field of research with many recent breakthroughs and many unexplored areas of study. The International Weed Genomics Consortium (IWGC) started in 2021 to address the roadblocks listed above and to promote the study of weedy plants. The IWGC is an open collaboration among academic, government, and industry researchers focused on producing genomic tools for weedy species from around the world. Through this collaboration, our initial aim is to provide chromosome-level reference genome assemblies for at least 50 important weedy species from across the globe that are chosen based on member input, economic impact, and global prevalence (Fig. 1). Each genome will include annotation of gene models and repetitive elements and will be freely available through public databases with no intellectual property restrictions. Additionally, future funding of the IWGC will focus on improving gene annotations and supplementing these reference genomes with tools that increase their utility.

### Reference genomes and data analysis tools

The first objective of the IWGC is to provide high-quality genomic resources for agriculturally important weeds. The IWGC therefore created two main resources for information about, access to, or analysis of weed genomic data (Fig. 1). The IWGC website (available at [167]) communicates the status and results of genome sequencing projects, information on training and funding opportunities, upcoming events, and news in weed genomics. It also contains details of all sequenced species including genome size, ploidy, chromosome number, herbicide resistance status, and reference genome assembly statistics. The IWGC either compiles existing data on genome size, ploidy, and chromosome number, or obtains the data using flow cytometry and cytogenetics (Fig. 1; Additional File 2: Fig S1-S4). Through this website, users can request an account to access our second main resource, an online genome database called WeedPedia (accessible at [168]), with an account that is created within 3–5 working days of an account request submission. WeedPedia hosts IWGC-generated and other relevant publicly accessible genomic data as well as a suite of bioinformatic tools. Unlike what is available for other fields, weed science did not have a centralized hub for genomics information, data, and analysis prior to the IWGC. Our intention in creating WeedPedia is to encourage collaboration and equity of access to information across the research community. Importantly, all genome assemblies and annotations from the IWGC (Table 1), along with the raw data used to produce them, will be made available through NCBI GenBank. Upon completion of a 1-year sponsoring member data confidentiality period for each species (dates listed in Table 1), scientific teams within the IWGC produce the first genome-wide investigation to submit for publication including whole genome level analyses on genes, gene families, and repetitive sequences as well as comparative analysis with other species. Genome assemblies and data will be publicly available through NCBI as part of these initial publications for each species.

WeedPedia is a cloud-based omics database management platform built from the software “CropPedia” and licensed from KeyGene (Wageningen, The Netherlands). The interface allows users to access, visualize, and download genome assemblies along with structural and functional annotation. The platform includes a genome browser, comparative map viewer, pangenome tools, RNA-sequencing data visualization tools, genetic mapping and marker analysis tools, and alignment capabilities that allow searches by keyword or sequence. Additionally, genes encoding known target sites of herbicides have been specially annotated, allowing users to quickly identify and compare these genes of interest. The platform is flexible, making it compatible with future integration of other data types such as epigenetic or proteomic information. As an online platform with a graphical user interface, WeedPedia provides user-friendly, intuitive tools that encourage users to integrate genomics into their research while also allowing more advanced users to download genomic data to be used in custom analysis pipelines. We aspire for WeedPedia to mimic the success of other public genomic databases such as NCBI, CoGe, Phytozome, InsectBase, and Mycocosm to name a few. WeedPedia currently hosts reference genomes for 40 species (some of which are currently in their 1-year confidentiality period) with additional genomes in the pipeline to reach a currently planned total of 55 species (Table 1). These genomes include both de novo reference genomes generated or in progress by the IWGC (31 species; Table 1), and publicly available genome assemblies of 24 weedy or related species that were generated by independent research groups (Table 2). As of May 2024, WeedPedia has over 370 registered users from more than 27 countries spread across 6 continents.

**Table 2:** Genome assemblies and genomic information for 24 weed species produced by other groups independently of the International Weed Genomics Consortium. Haploid (1n) genome size estimations are either calculated through flow cytometry or *k*-mer estimation

Scientific name	Common name	*x*	*n*	1n genome size estimate (Gbp)	Genome assembly size (Gbp)
*Alopecurus myosuroides*	Blackgrass	7	7	3.56 [72]	3.4–3.56 [72, 161]
*Bassia scoparia*	Kochia	9	9	0.969 [169]	0.970 [169]
*Bromus tectorum*	Cheatgrass	7	7		2.48 [88]
*Chenopodium formosanum* (domesticated genotype of *C. album*)	Djulis	9	27	1.69 [170]	1.59 [170]
*Chloris virgata*	Feather finger grass	20	20		0.336 [171]
*Conyza canadensis* (*Erigeron canadensis*)	Horseweed	9	9	0.425 [172]	0.426 [172]
*Echinochloa colona* (crop genotype)	Junglerice	9	27	1.18 [76]	1.13 [76]
*Echinochloa crus-galli*	Barnyardgrass	9	27	1.4 [173]	1.34 [76]
*Echinochloa oryzicola* (syn. *E. phyllopogon*)	Late watergrass	9	18	1.0 [173]	0.95 [76] 1.0 [174]
*Eleusine indica*	Goosegrass	9	9		0.51 [156]
*Ipomoea purpurea*	Common morning-glory	15	15	0.81 [122]	0.60 [122]
*Lolium perenne*	Perennial ryegrass	7	7		2.63 (Bushman and Robbins, pers. comm.)
*Leersia perrieri*	Cutgrass	12	12		0.267 [175]
*Oryza sativa f. spontanea*	Weedy rice	12	12		0.37 [176]
*Panicum miliaceum*	Wild proso millet	9	18		0.85 [177]
*Poa annua*	Annual bluegrass	7	14	1.78 [178]	1.89 [129]
*Poa infirma*	Early meadow-grass	7	7	1.17 [179]	1.13 [179]
*Poa supina*	Supine bluegrass	7	7	0.66 [179]	0.64 [179]
*Poa trivialis*	Roughstalk bluegrass	7	7		1.35 [180]
*Pueraria montana* var. *lobata*	Kudzu	11	11		0.98 [181]
*Setaria viridis*	Green foxtail	9	9	0.40 [182]	0.40 [182]
*Striga asiatica*	Red witchweed	12	12	0.6 [183]	0.47 [183]
*Striga hermonthica*	Purple witchweed	10	20	1.48 [184]	0.96 [184]
*Thlaspi arvensis*	Field pennycress	7	7	0.5 [185]	0.53 [185]

The IWGC reference genomes are generated in partnership with the Corteva Agriscience Genome Center of Excellence (Johnston, Iowa) using a combination of single-molecule long-read sequencing, optical genome maps, and chromosome conformation mapping. This strategy has already yielded highly contiguous, phased, chromosome-level assemblies for 26 weed species, with additional assemblies currently in progress (Table 1). The IWGC assemblies have been completed as single or haplotype-resolved double-haplotype pseudomolecules in inbreeding and outbreeding species, respectively, with multiple genomes being near gapless. For example, the de novo assemblies of the allohexaploids *Conyza sumatrensis* and *Chenopodium album* have all chromosomes captured in single scaffolds and most chromosomes being gapless from telomere to telomere. Complementary full-length isoform (IsoSeq) sequencing of RNA collected from diverse tissue types and developmental stages assists in the development of gene models during annotation.

As with accessibility of data, a core objective of the IWGC is to facilitate open access to sequenced germplasm when possible for featured species. Historically, the weed science community has rarely shared or adopted standard germplasm (e.g., specific weed accessions). The IWGC has selected a specific accession of each species for reference genome assembly (typically susceptible to herbicides). In collaboration with a parallel effort by the Herbicide Resistant Plants committee of the Weed Science Society of America, seeds of the sequenced weed accessions will be deposited in the United States Department of Agriculture Germplasm Resources Information Network [186] for broad access by the scientific community and their accession numbers will be listed on the IWGC website. In some cases, it is not possible to generate enough seed to deposit into a public repository (e.g., plants that typically reproduce vegetatively, that are self-incompatible, or that produce very few seeds from a single individual). In these cases, the location of collection for sequenced accessions will at least inform the community where the sequenced individual came from and where they may expect to collect individuals with similar genotypes. The IWGC ensures that sequenced accessions are collected and documented to comply with the Nagoya Protocol on access to genetic resources and the fair and equitable sharing of benefits arising from their utilization under the Convention on Biological Diversity and related Access and Benefit Sharing Legislation [187]. As additional accessions of weed species are sequenced (e.g., pangenomes are obtained), the IWGC will facilitate germplasm sharing protocols to support collaboration. Further, to simplify the investigation of herbicide resistance, the IWGC will link WeedPedia with the International Herbicide-Resistant Weed Database [104], an already widely known and utilized database for weed scientists.

### Training and collaboration in weed genomics

Beyond producing genomic tools and resources, a priority of the IWGC is to enable the utilization of these resources across a wide range of stakeholders. A holistic approach to training is required for weed science generally [188], and we would argue even more so for weed genomics. To accomplish our training goals, the IWGC is developing and delivering programs aimed at the full range of IWGC stakeholders and covering a breadth of relevant topics. We have taken care to ensure our approaches are diverse as to provide training to researchers with all levels of existing experience and differing reasons for engaging with these tools. Throughout, the focus is on ensuring that our training and outreach result in impacts that benefit a wide range of stakeholders.

Although recently developed tools are incredibly enabling and have great potential to replace antiquated methodology [189] and to solve pressing weed science problems [14], specialized computational skills are required to fully explore and unlock meaning from these highly complex datasets. Collaboration with, or training of, computational biologists equipped with these skills and resources developed by the IWGC will enable weed scientists to expand research programs and better understand the genetic underpinnings of weed evolution and herbicide resistance. To fill existing skill gaps, the IWGC is developing summer bootcamps and online modules directed specifically at weed scientists that will provide training on computational skills (Fig. 1). Because successful utilization of the IWGC resources requires more than general computational skills, we have created three targeted workshops that teach practical skills related to genomics databases, molecular biology, and population genomics (available at [190]). The IWGC has also hosted two official conference meetings, one in September of 2021 and one in January of 2023, with more conferences planned. These conferences have included invited speakers to present successful implementations of weed genomics, educational workshops to build computational skills, and networking opportunities for research to connect and collaborate.

Engagement opportunities during undergraduate degrees have been shown to improve academic outcomes [191, 192]. As one activity to help achieve this goal, the IWGC has sponsored opportunities for US undergraduates to undertake a 10-week research experience, which includes an introduction to bioinformatics, a plant genomics research project that results in a presentation, and access to career building opportunities in diverse workplace environments. To increase equitable access to conferences and professional communities, we supported early career researchers to attend the first two IWGC conferences in the USA as well as workshops and bootcamps in Europe, South America, and Australia. These hybrid or in-person travel grants are intentionally designed to remove barriers and increase participation of individuals from backgrounds and experiences currently underrepresented within weed/plant science or genomics [193]. Recipients of these travel awards gave presentations and gained the measurable benefits that come from either virtual or in-person participation in conferences [194]. Moving forward, weed scientists must amass skills associated with genomic analyses and collaborate with other area experts to fully leverage resources developed by the IWGC.

The tools generated through the IWGC will enable many new research projects with diverse objectives like those listed above. In summary, contiguous genome assemblies and complete annotation information will allow weed scientists to join plant breeders in the use of genetic mapping for many traits including stress tolerance, plant architecture, and herbicide resistance (especially important for cases of NTSR). These assemblies will also allow for investigations of population structure, gene flow, and responses to evolutionary mechanisms like genetic bottlenecking and artificial selection. Understanding gene sequences across diverse weed species will be vital in modeling new herbicide target site proteins and designing novel effective herbicides with minimal off-target effects. The IWGC website will improve accessibility to weed genomics data by providing a single hub for reference genomes as well as phenotypic and genotypic information for accessions shared with the IWGC. Deposition of sequenced germplasm into public repositories will ensure that researchers are able to access and utilize these accessions in their own research to make the field more standardized and equitable. WeedPedia allows users of all backgrounds to quickly access information of interest such as herbicide target site gene sequence or subcellular localization of protein products for different genes. Users can also utilize server-based tools such as BLAST and genome browsing similar to other public genomic databases. Finally, the IWGC is committed to training and connecting weed genomicists through hosting trainings, workshops, and conferences.

## Conclusions

Weeds are unique and fascinating plants, having significant impacts on agriculture and ecosystems; and yet, aspects of their biology, ecology, and genetics remain poorly understood. Weeds represent a unique area within plant biology, given their repeated rapid adaptation to sudden and severe shifts in the selective landscape of anthropogenic management practices. The production of a public genomics database with reference genomes and annotations for over 50 weed species represents a substantial step forward towards research goals that improve our understanding of the biology and evolution of weeds. Future work is needed to improve annotations, particularly for complex gene families involved in herbicide detoxification, structural variants, and mobile genetic elements. As reference genome assemblies become available; standard, affordable methods for gathering genotype information will allow for the identification of genetic variants underlying traits of interest. Further, methods for functional validation and hypothesis testing are needed in weeds to validate the effect of genetic variants detected through such experiments, including systems for transformation, gene editing, and transient gene silencing and expression. Future research should focus on utilizing weed genomes to investigate questions about evolutionary biology, ecology, genetics of weedy traits, and weed population dynamics. The IWGC plans to continue the public–private partnership model to host the WeedPedia database over time, integrate new datasets such as genome resequencing and transcriptomes, conduct trainings, and serve as a research coordination network to ensure that advances in weed science from around the world are shared across the research community (Fig. 1). Bridging basic plant genomics with translational applications in weeds is needed to deliver on the potential of weed genomics to improve weed management and crop breeding.

### Supplementary Information

Additional file 1. List of completed and in-progress genome assemblies of weed species pollinated by insects (Table S1).

Additional file 2. Methods and results for visualizing and counting the metaphase chromosomes of hexaploid* Avena fatua *(Fig S1); diploid *Lolium rigidum *(Fig S2); tetraploid *Phalaris minor *(Fig S3); and tetraploid *Salsola tragus *(Fig S4).

Additional file 3. Review history.

## Data Availability

All genome assemblies and related sequencing data produced by the IWGC will be available through NCBI as part of publications reporting the first genome-wide analysis for each species.
