# Supplementary material for: Current status of community resources and priorities for weed genomics research
Source: Genome Biol. 2024 May 27;25:139. doi: 10.1186/s13059-024-03274-y (PMC11129445; doi:10.1186/s13059-024-03274-y)
Supplement: Supplementary file 1 — Additional file 1. List of completed and in-progress genome assemblies of weed species pollinated by insects (Table S1). [file 13059_2024_3274_MOESM1_ESM.docx]

Table S1. Completed and in-progress genome assemblies of weed species pollinated by insects.

| **Weed species** | **Family** | **Entomophilous pollination** | **Reference** |
| --- | --- | --- | --- |
| *Cirsium arvense* | Asteraceae | Yes | [1] |
| *Conyza canadensis* | Asteraceae | Primarily self-pollinating, although insects were observed visiting flowers | [2] |
| *Erigeron sumatrensis* | Asteraceae | Yes | [3] |
| *Parthenium hysterophorus* | Asteraceae | Yes | [4, 5] |
| *Raphanus raphanistrum* | Brassicaceae | Yes | [6, 7] |
| *Salsola tragus* | Chenopodiaceae | Mainly anemophilous but also visited by insects | [8] |
| *Convolvulus arvensis* | Convolvulaceae | Yes | [9] |
| *Ipomoea purpurea* | Convolvulaceae | Yes | [10] |
| *Euphorbia esula* | Euphorbiaceae | Yes | [11] |
| *Euphorbia heterophylla* | Euphorbiaceae | Yes | [12] |
| *Verbascum blattaria* | Scrophulariaceae | Yes | [13] |

**References**

1. Tiley GED. Biological Flora of the British Isles: *Cirsium arvense* (L.) Scop. J Ecol*.* 2010;98(4):938-83.

2. Weaver SE. The biology of Canadian weeds. 115. *Conyza canadensis*. Can J Plant Sci*.* 2001;81(4):867-75.

3. Hao J-H, Qiang S, Liu Q-Q, Cao F. Reproductive traits associated with invasiveness in *Conyza sumatrensis*. J Syst Evol*.* 2009;47(3):245-54.

4. Ojija F, Arnold SEJ, Treydte AC. Impacts of alien invasive *Parthenium hysterophorus* on flower visitation by insects to co-flowering plants. Arthropod Plant Interact*.* 2019;13(5):719-34.

5. Usharani B, Solomon Raju AJ. Reproductive ecology of the globally invasive whitetop weed, *Parthenium hysterophorus* (Asteraceae). Phytol Balc*.* 2018;24):225-38.

6. Conner JK, Davis R, Rush S. The effect of wild radish floral morphology on pollination efficiency by four taxa of pollinators. Oecologia*.* 1995;104(2):234-45.

7. Divija SD, Kamala Jayanthi PD, Varun YB, Saravan Kumar P, Krishnarao G, Nisarga GS. Diversity, abundance and foraging behaviour of insect pollinators in Radish (*Raphanus raphanistrum* subsp. *sativus* L.). J Asia Pac Entomol*.* 2022;25(2):101909.

8. Larson DL, Larson JL, Symstad AJ, Buhl DA, Portman ZM. Coflowering invasive plants and a congener have neutral effects on fitness components of a rare endemic plant. Ecol Evol*.* 2021;11(9):4750-62.

9. Sosnoskie LM, Hanson BD, Steckel LE. Field bindweed (*Convolvulus arvensis*): “all tied up”. Weed Technol*.* 2020;34(6):916-21.

10. Liu CC, Gui MY, Sun YC, Wang XF, He H, Wang TX, et al. Doubly guaranteed mechanism for pollination and fertilization in *Ipomoea purpurea*. Plant Biol*.* 2020;22(5):910-16.

11. Montgomery BR. Pollination of *Sisyrinchium campestre* (Iridaceae) in Prairies Invaded by the Introduced Plant *Euphorbia esula* (Euphorbiaceae). Am Midl Nat*.* 2009;162(2):239-52.

12. Narbona E, Ortiz PL, Arista M. Functional andromonoecy in *Euphorbia* (Euphorbiaceae). Ann Bot*.* 2002;89(5):571-7.

13. Gross KL, Werner PA. The biology of Canadian weeds: 28. *Verbascum thapsus* L. and *V. blattaria* L. Can J Plant Sci*.* 1978;58:401-13.
