# Supplementary material for: Current status of community resources and priorities for weed genomics research
Source: Genome Biol. 2024 May 27;25:139. doi: 10.1186/s13059-024-03274-y (PMC11129445; doi:10.1186/s13059-024-03274-y)
Supplement: Supplementary file 2 — Additional file 2. Methods and results for visualizing and counting the metaphase chromosomes of hexaploid Avena fatua (Fig S1); diploid Lolium rigidum (Fig S2); tetraploid Phalaris minor (Fig S3); and tetraploid Salsola tragus (Fig S4). [file 13059_2024_3274_MOESM2_ESM.docx]

Additional File 2. Methods and results for visualizing and counting the metaphase chromosomes of hexaploid *Avena fatua*; diploid *Lolium rigidum*; tetraploid *Phalaris minor*; and tetraploid *Salsola tragus*.

**Preparation of metaphase chromosomes:**

Young actively growing root tips were collected early in the morning (before 8 am) and treated with N_2_O gas at 10 bar for 3 h, followed by a pre-treatment with α-bromonapthalene for 8 h at 4 °C, and finally, overnight fixation in 3:1 (ethanol: acetic acid) solution at room temperature. The root tips were incubated in a humid chamber with an enzyme mixture containing 0.3% pectolyase, 0.2% cellulase, and 0.2% cytohelicase for 45 min at 37 °C. To acquire well-spread metaphase chromosomes, the macerated root tips were split and squashed with 65% acetic acid (20 μl/slide). The slides were subsequently stored at -80 °C until further use. Vectashield solution diluted with 2% 4, 6-diamidino-2-phenylindole (DAPI) was used to observe metaphase chromosomes under the microscope. All the images were captured at 60× emulsion oil magnification using an Olympus BX61 fluorescent microscope equipped with a Hamamatsu C10600 camera and further processed with the MetaMorph software.


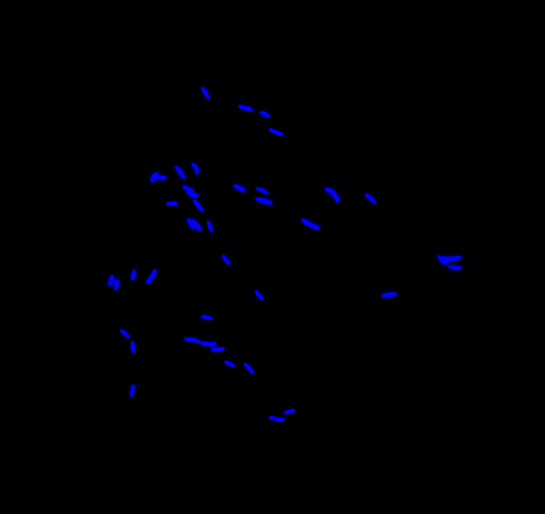

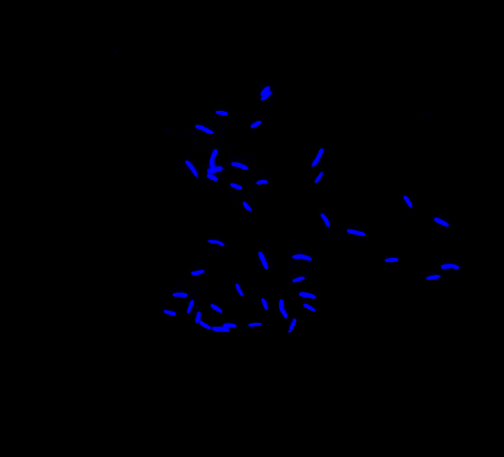
Fig S1. Metaphase chromosomes of hexaploid *Avena fatua.*

2n = 6x = 42

**Wild Oat (*Avena fatua)***

Fig S2. Metaphase chromosomes of diploid *Lolium rigidum.*

**Annual ryegrass *(Lolium rigidum)***


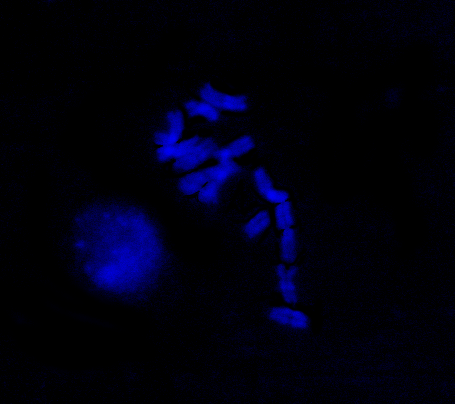

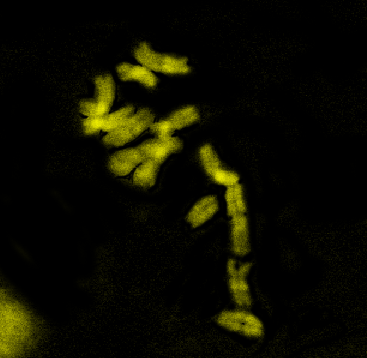
Fig S3. Metaphase chromosomes of tetraploid *Phalaris minor.*
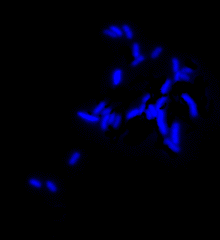

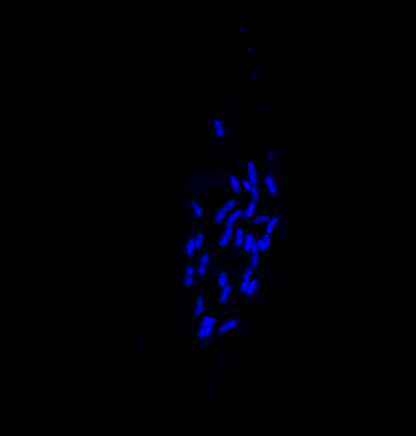

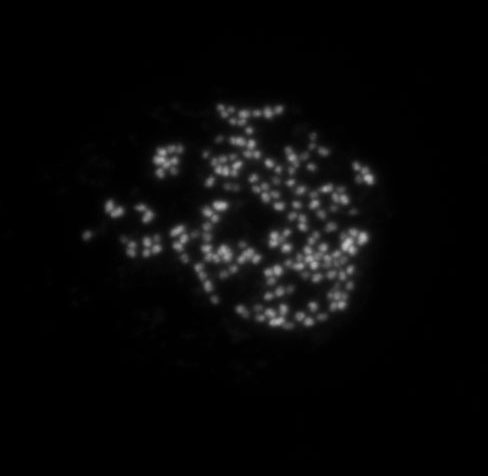


2n = 2x = 14

**Little seed canary grass *(Phalaris minor)***

2n =4x = 28

Fig S4. Metaphase chromosomes of tetraploid *Salsola tragus.*


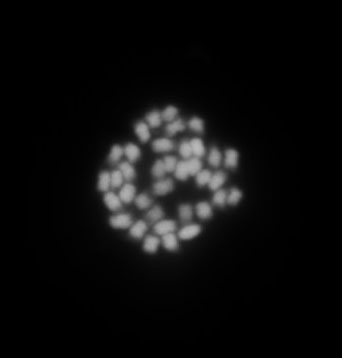

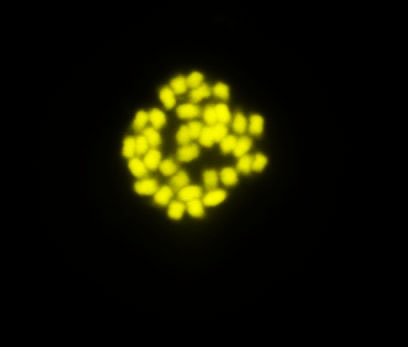

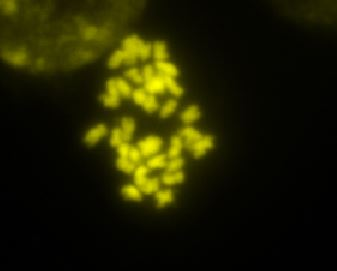


**Russian thistle (*Salsola tragus*)**

2n = 4x = 36
