# Supplementary material for: Current status of community resources and priorities for weed genomics research
Source: Genome Biol. 2024 May 27;25:139. doi: 10.1186/s13059-024-03274-y (PMC11129445; doi:10.1186/s13059-024-03274-y)
Supplement: Supplementary file 3 — Additional file 3. Review history. [file 13059_2024_3274_MOESM3_ESM.docx]

Review History

**First round of review**

**Reviewer 1**

**Were you able to assess all statistics in the manuscript, including the appropriateness of statistical tests used?**

Yes.

**Comments to author:**

This manuscript describes the resources available through the IWGC website, and discusses how these resources can be used to answer critical questions and solve significant problems associated with weed genomics. The only criticism I have is that often throughout the manuscript, the specific tools and resources' available at the IWGC website were not identified after each problem/research direction was described. Also, I think a section on the use of GWAS to identify NTSR mechanisms (and really to confirm the role of TSR genes in the resistance mechanisms prorogating or evolving in a given population should get more attention... and as noted, provided with examples of how the resources at the IWGC website could assist in such studies. Otherwise there were just a few minor suggested changes as noted in the marked up manuscript (hopefully attached).

**Reviewer 2**

**Were you able to assess all statistics in the manuscript, including the appropriateness of statistical tests used?**

Yes.

**Comments to author:**

This manuscript is neither an original research paper nor a review paper, although the second half of the ms has aspects of a review paper. The main goal of the ms appears to describe a consortium that would mainly be of interest to weed scientists. It has some grant proposal-like wording. Thus, for that goal, the writing appears to be adequate and appropriate.

Below are a few minor comments.

Abstract evolutionary mechanisms appears to be a topic of interest, but is absent from the abstract

Line 57 weeds are not systems: reword

Line 87 if the list of authors comprises is the consortium, there appears to be no government representatives

Line 119 url for weedpedia would be helpful

Line 203 where? In the USA? REU is an NSF term

**Authors Response**

**Point-by-point responses to the reviewers’ comments:**

*Response:*

*We responded to all Reviewer 1 comments and suggestions to highlight specific tools and resources from the IWGC, and added a segment about GWAS for NTSR studies.*

*In response to Reviewer 2, we have restructured the manuscript as a typical review, so that the initial emphasis is on the review of the field and the IWGC is introduced later as a response to the needs identified in the review. We have also rewritten the abstract to reflect the structure as a review and to include evolutionary mechanisms.*

Reviewer #1: This manuscript describes the resources available through the IWGC website, and discusses how these resources can be used to answer critical questions and solve significant problems associated with weed genomics. The only criticism I have is that often throughout the manuscript, the specific tools and resources' available at the IWGC website were not identified after each problem/research direction was described. Also, I think a section on the use of GWAS to identify NTSR mechanisms (and really to confirm the role of TSR genes in the resistance mechanisms prorogating or evolving in a given population should get more attention... and as noted, provided with examples of how the resources at the IWGC website could assist in such studies. Otherwise there were just a few minor suggested changes as noted in the marked up manuscript (hopefully attached).

*Response: We thank the reviewer for these observations. We have specified the specific tools and resources available at the IWGC website on lines 397-407. The use of GWAS for understanding NTSR is specifically discussed on lines 267-271.*

Reviewer #1 comments in marked up manuscript PDF:

Lines 73-74: this statement is debatable as per the previous paragraph

*Response: Agreed, and we deleted this statement*

Line 75: insert ‘and epigenetic’ after evolutionary

*Response: Agreed and inserted.*

Line 75: delete ‘rescue’, replace with ‘response’

*Response: We think evolutionary rescue is an important concept to keep, specifically that weeds are small populations able to survive in the face of near catastrophic population collapses*

Line 112: add URL link to IWGC website in addition to the citation

The journal guidelines suggest citing websites as references and providing the URL in the References section. We have changed the in-text citation to be more clear that the URL is in the Reference list.

Line 143: I just checked and the number is nowhere near 40 (more like 27). I was looking for kochia as I know it is done, but it was not downloadable.

*Response: The number of 40 is correct as it includes genomes that are in the confidentiality time period for sponsoring members along with public genomes available to all users; both confidential and public genomes are hosted at WeedPedia. A public user account, such as the one likely used by the reviewer, assuming they are not an employee of a sponsoring company, would see the number of 27. The genomes in confidentiality period will be publicly available soon, most within the next 6 months. Kochia is currently available to download; it is listed under Bassia scoparia.*

Line 148: delete ‘representing’ and replace with ‘from’

*Response: Done as requested.*

Line 167-170: Name them here and/or provide a table; and How does this work for weeds that have poor seed production from specific accessions when there are issues of self incompatibility? Or from weeds that don't produce seeds at all but rather are exclusively propagated vegetatively (like alligatorweed for example?)

*Response: Germplasm accessibility is now discussed in greater depth with some of these challenges addressed on lines 432-441. Many of the seed stores have not been deposited, so instead of providing a table that will soon be defunct, we point readers to the IWGC website, which will have GRIN accession numbers updated as they become available.*

Line 200: include URL link to IWGC website training page

*Response: As before, we have made it more clear that the URL for the website is in the References section as per journal guidelines.*

Line 225-227: Could note specifically how the IWGC is facilitating this sort of research

*Response: The outcomes of the IWGC and specifically how they are enabling/facilitating research are now more explicitly recapped in lines 490-507. IWGC conferences make great opportunities for researchers to connect to experts in their field and form collaborations.*

Line 236-238: Only if the IWGC plans on developing genotypes for diverse and representative populations.... OK just got to the next paragraph :-) never mind.

*Response: We hope that as accessions are sequenced/phenotyped that researchers make their data available. We will try to keep up with new publications to include accession numbers of publicly available resources in each species’ page on the IWGC website.*

Lines 261-263: Again, provide information on how, specifically, the IWGC plans to assist with solving this problem. Will there be a protocol page or a place to order vectors of interest?

*Response: this is a call for future work; IWGC will facilitate, but we hope funding agencies see this as a call for action; the IWGC does not have current plans to make these protocols or create vectors, etc.*

Line 277: Such studies will be facilitated by the IWGC through the cross referencing of papers and key word search capabilities present on the IWGC website (or something like that). If those capabilities don't exist (yet) note any plans to make them available.

*Response: The IWGC has no plans to create a database of scientific papers, but notable publications will be featured as news updates (similar to how journals tweet about high-impact articles).*

Line 336: again reinforce how the IWGC resources assist in such studies.

*Response: The existence of a reference genome in these species enables such studies (or at least allows for more robust methods). We have adapted the paper to lead with research areas and then introduce the IWGC later. Specific resources generated by the consortium and how they impact the field are now more explicitly listed on lines 490-507.*

Line 398: Missed the opportunity for using GWAS approaches for mapping NTSR loci using genome resources at the IWGC.

*Response: This is now more specifically discussed on lines 267-271.*

Line 450: insert “such as those provided and facilitated by the IWGC.” at the end of the sentence

*Response: Because we have restructured the paper to introduce the IWGC at the end, this reference would be out of place. We discuss the role of the IWGC in the development of resources and the facilitation of collaboration in the later section of the paper.*

Line 465: delete s from genomics and insert this after genomic “resources such as those provided by the IWGC”

*Response: Done as requested.*

Reviewer #2: This manuscript is neither an original research paper nor a review paper, although the second half of the ms has aspects of a review paper. The main goal of the ms appears to describe a consortium that would mainly be of interest to weed scientists. It has some grant proposal-like wording. Thus, for that goal, the writing appears to be adequate and appropriate.

Below are a few minor comments.

*Response: We thank the reviewer for this insight. We have restructured the manuscript as a typical review, with overviews of the field and specific applications of genomics to weed science questions. We then introduce the IWGC as a response to meet the identified needs in the review, and describe the resources available.*

Abstract: evolutionary mechanisms appears to be a topic of interest, but is absent from the abstract

*Response: We have changed the abstract to be more reflective of the new structure of the paper and have included mention of evolutionary mechanisms.*

Line 57 weeds are not systems: reword

*Response: this has been changed to models*

Line 87 if the list of authors comprises is the consortium, there appears to be no government representatives

*Response: The list of authors is not a complete listing of consortium members. Government representatives have WeedPedia accounts; submit data (reference genomes) to share, including examples of using WeedPedia as an accessible data repository during publication submission/review; collaborate on new genome assembly; and attend conferences and training workshops. We prefer to keep government researchers listed, although none are co-authors on this manuscript.*

Line 119 url for weedpedia would be helpful

*Response: This URL is now provided as a reference as per the journal guidelines, and the in-text citation points readers to this reference.*

Line 203 where? In the USA? REU is an NSF term

*Response: We rewrote and clarified this sentence, indicating that this is an opportunity for US students and removing the REU abbreviation.*

*Additional changes that we made in this revision:*

*Line 381-384: added this sentence in response to editor feedback*

*Through this website, users can request an account to access our second main resource, an online genome database called WeedPedia (https://weedpedia.weedgenomics.org/), with an account that is created within 3-5 working days of an account request submission.*

*One additional published weed genome added to table 1:*

*Cyperus esculentus, Zhao et al 2023*

**Second round of review**

**Reviewer 1**

The authors have adequately addressed all of my concerns. The manuscript is suitable for publication.
